# A Novel Aniline Derivative from *Peganum harmala* L. Promoted Apoptosis via Activating PI3K/AKT/mTOR-Mediated Autophagy in Non-Small Cell Lung Cancer Cells

**DOI:** 10.3390/ijms241612626

**Published:** 2023-08-10

**Authors:** Zhongnan Wu, Wen Li, Qing Tang, Laiqiang Huang, Zhaochun Zhan, Yaolan Li, Guocai Wang, Xiaoyong Dai, Yubo Zhang

**Affiliations:** 1 Guangdong Clinical Translational Center for Targeted Drug, Department of Pharmacology, School of Medicine, Jinan University, Guangzhou 510632, China; 2 College of Pharmacy, Guangdong Medical University, Dongguan 523808, China; 3 Institute of Traditional Chinese Medicine & Natural Products, Guangdong Province Key Laboratory of Pharmacodynamic Constituents of TCM and New Drugs Research, College of Pharmacy, Jinan University, Guangzhou 510632, Chinatwangguocai@jnu.edu.cn (G.W.); 4 Institute of Biopharmaceutical and Health Engineering, Shenzhen Key Laboratory of Gene and Antibody Therapy, State Key Laboratory of Chemical Oncogenomics, Shenzhen International Graduate School, Tsinghua University, Shenzhen 518055, China

**Keywords:** aniline derivative, CD133, autophagy, PI3K/AKT/mTOR, apoptosis, NSCLC

## Abstract

Non-small cell lung cancer (NSCLC) is a common clinical malignant tumor with limited therapeutic drugs. Leading by cytotoxicity against NSCLC cell lines (A549 and PC9), bioactivity-guided isolation of components from *Peganum harmala* seeds led to the isolation of pegaharoline A (PA). PA was elucidated as a structurally novel aniline derivative, originating from tryptamine with a pyrrole ring cleaved and the degradation of carbon. Biological studies showed that PA significantly inhibited NSCLC cell proliferation, suppressed DNA synthesis, arrested the cell cycle, suppressed colony formation and HUVEC angiogenesis, and blocked cell invasion and migration. Molecular docking and surface plasmon resonance (SPR) demonstrated PA could bind with CD133, correspondingly decreased CD133 expression to activate autophagy via inhibiting the PI3K/AKT/mTOR pathway, and increased ROS levels, Bax, and cleaved caspase-3 to promote apoptosis. PA could also decrease p-cyclinD1 and p-Erk1/2 and block the EMT pathway to inhibit NSCLC cell growth, invasion, and migration. According to these results, PA could inhibit NSCLC cell growth by blocking PI3K/AKT/mTOR and EMT pathways. This study provides evidence that PA has a promising future as a candidate for developing drugs for treating NSCLC.

## 1. Introduction

Lung cancer has a high prevalence and fatality rate because of its malignant characteristics [1]. Among them, NSCLC makes up around 80% to 85% of all reported cases of lung cancer [2]. Typically, antineoplastic medications such as paclitaxel, docetaxel, and gemcitabine are utilized as the standard treatment for NSCLC [3]. However, the excessive utilization of cytotoxic drugs may result in the development of drug-resistant strains and could potentially diminish the effectiveness of current treatment medications [4,5,6]. As a result, there is a need to explore new alternative molecules that can address these limitations. Over the last few years, an increasing number of natural compounds, such as alkaloids, flavonoids, and phenolic acid derivatives, have been identified for their potent cytotoxic effects on lung cancer [7,8]. Therefore, finding novel molecules with therapeutic potential for NSCLC from plants offers a promising avenue for future research and development.

Cancer stem cells (CSCs) are known for their significant capabilities of proliferation, dissemination, and treatment resistance. They express various CD antigens, such as CD44, CD90, CD117, CD133, and CD166 [9,10]. CD133 is commonly utilized for the identification of CSCs in lung cancer [11]. Despite its widespread use, the exact role of CD133 in normal lung cancer cells remains largely unknown. Some research groups have confirmed that CD133 may have a function in promoting survival and inhibiting apoptosis in CSCs [12]. Wang et al. found that there was a strong association between CD133 and an unfavorable prognosis for NSCLC patients. As a result, CD133 may be proven as a valuable prognosticator for such patients [13]. Huang et al. utilized nanomicelles that were conjugated with CD133 and CD44 aptamers and loaded with gefitinib in order to target lung cancer cells. Their findings demonstrated that these nanomicelles exhibited superior therapeutic effectiveness against lung-cancer-initiating cells when compared to non-targeted and single-target nanomicelles [14]. These findings indicate that CD133 could be a promising target in cancer treatments.

Autophagy and apoptosis are essential mechanisms that contribute significantly to the progression of lung cancer. Autophagy is a cellular process in eukaryotes that regulates homeostasis by removing degradation products through lysosomal compartments [15]. Autophagy is regulated by multiple systems, including the ULK1, VPS34, LC3-II, and the process of ubiquitination, all of which are essential for the different phases of autophagy [16]. Apoptosis is extensively studied as it is primarily accountable for the death of cells. This process is regulated by signals from both inside and outside the cell. Apoptosis triggers various structural alterations in a cell, including the fragmentation and condensation of the nucleus, the permeabilization of MOMP, the creation of apoptotic fragments, the establishment of membrane blebs, and the shrinking of the cell [17]. Numerous studies have documented that certain natural products can impact the autophagy and apoptosis pathways, displaying potential anti-cancer capabilities [18].

*Peganum harmala* L. is a type of plant that can be found in desert regions and arid grasslands of Asia, Africa, and the Mediterranean, and belongs to a botanical family known as Zygophyllaceae [19,20]. In China, the seeds of *P. harmala* have been recognized as “Luo-tuo-peng-zi” and have been utilized to treat various illnesses, including asthma, cough, and rheumatism [20]. Alkaloids were reported to be the main bioactive components of this plant, with diverse bioactivities of antitumor [21,22], anti-inflammatory [23,24], antiviral [25,26,27], antioxidant activities [28,29], etc. During our investigation into antitumor compounds found in Chinese medicinal herbs, we discovered that the alkaloids extracted from *P. harmala* seeds exhibited specific cytotoxic effects on A549 and PC9 NSCLC cell lines, with IC_50_ values of 18.92 ± 0.60 μg/mL and 21.20 ± 0.90 μg/mL, respectively. Further analysis of the total alkaloids resulted in the identification of a novel tryptamine-derived alkaloid, named pegaharoline A (PA). PA exhibited strong antitumor activity against NSCLC cells (A549: IC_50_ = 2.39 ± 0.27 µM; PC9: IC_50_ = 3.60 ± 0.41 µM). Further biological studies showed that PA could bind with CD133 and decrease CD133 expression to inhibit NSCLC cell growth via activating autophagy and cell apoptosis.

## 2. Results

### 2.1. Structural Elucidation of Pegaharoline A (PA)

**Figure 1:**
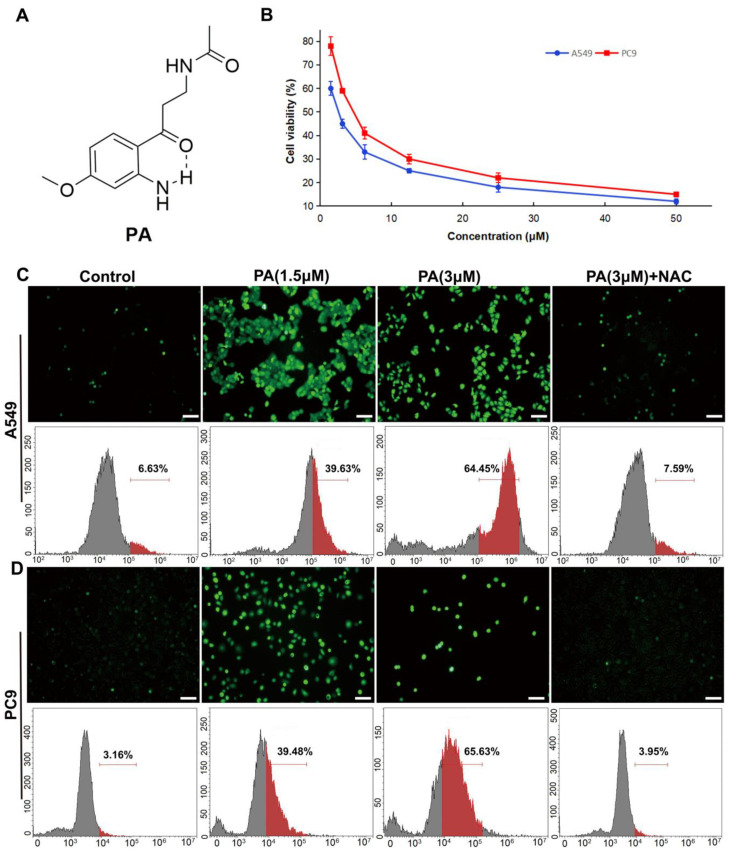
PA (pegaharoline A) inhibited the proliferation of NSCLC cell lines (A549 and PC9) through promoting the production of ROS. (**A**) Chemical structure of PA. (**B**) The inhibition ratios of A549 and PC9 cells were detected by MTT assay at 48 h after treatment with various concentrations of PA. The DCFH-DA fluorescence probe was used for detecting ROS by using immunofluorescence and flow cytometry in PA-treated A549 cells (**C**) and PC9 cells (**D**) for 24 h, respectively. Scale bar 100 μm. Green dots in (**C**,**D**) (upper panel) indicated the ROS fluorescence-positive cells. Red parts in (**C**,**D**) (lower panel) indicated the rate of the ROS fluorescence-positive cells.

The [M + H]^+^ ion peak of pegaharoline A was observed at *m*/*z* 237.1233 (calcd. for C_12_H_17_N_2_O_3_ 237.1234), which is in agreement with the molecular formula of C_12_H_16_N_2_O_3_ (Figure S1). Based on the spectroscopic data of UV and IR, the compound presented absorption peaks at 205, 234, and 287 nm (Figure S2) and had an NH group at 3406 cm^−1^ (Figure S3). The ^1^H NMR spectrum (shown in Table S1) demonstrated the existence of an AMX aromatic ring (*δ*_H_ 7.64 (1H, d, *J* = 9.0 Hz), 6.24 (1H, d, *J* = 2.4 Hz), 6.14 (1H, dd, *J* = 9.0, 2.4 Hz)), a -CH_2_CH_2_- spin system (*δ*_H_ 3.32 (2H, t, *J* = 6.8 Hz), 2.98 (2H, t, *J* = 6.8 Hz)), a methoxyl (*δ*_H_ 3.73 (3H, s)), a methyl (*δ*_H_ 1.77 (3H, s)), an NH group (*δ*_H_ 7.89 (1H, t, *J* = 5.0 Hz)), and an amino group (*δ*_H_ 7.34 (2H, br s)) (Figure S4). The ^13^C NMR data (presented in Table S1) indicated the existence of 12 carbons, consisting of 2 carbonyl carbons (*δ*_C_ 198.4 and 169.3), 6 aromatic carbons (*δ*_C_ 163.8, 153.6, 133.3, 111.3, 103.6, and 98.5), 2 methylenes (*δ*_C_ 38.1 and 34.9), a methoxyl (*δ*_C_ 55.0) and a methyl (*δ*_C_ 22.6) (Figures S5 and S6). Pegaharoline A could be derived from the indole alkaloid peganumaline B [22] (Scheme S1, in Supporting Information). The main discrepancies observed between pegaharoline A and peganumaline B were the lack of a carbamate carbonyl (*δ*_C_ 179.5), as well as the absence of an oxygenated quaternary carbon (*δ*_C_ 74.1). However, pegaharoline A showed the presence of a keto carbonyl (*δ*_C_ 198.4) and a broad NH_2_ singlet. This implied that the pyrrole ring of peganumaline B was cleaved to afford an aniline derivative (PA) (Figure 1A). Moreover, the carbamate carbonyl was reduced and the oxygenated quaternary carbon was further oxidized to a keto carbonyl. The structural assignment of pegaharoline A was demonstrated by the ^1^H-^1^H COSY correlations of H-4 (*δ*_H_ 7.64)/H-5 (*δ*_H_ 6.14) and H-9 (*δ*_H_ 2.98)/H-10 (*δ*_H_ 3.32), together with the HMBC cross-peaks from H-4 to C-2 (*δ*_C_ 153.6)/C-3 (*δ*_C_ 111.3)/C-6 (*δ*_C_ 163.8)/C-8 (*δ*_C_ 198.4), from H-5 to C-3/C-7 (*δ*_C_ 98.5), from H-7 (*δ*_H_ 6.24) to C-3/C-5 (*δ*_C_ 103.6), from H-9 to C-8/C-10 (*δ*_C_ 34.9), from H-10 to C-8/C-9 (*δ*_C_ 38.1)/C-12 (*δ*_C_ 169.3), from H-13 (*δ*_H_ 1.77) to C-12, and from 6-OCH_3_ (*δ*_H_ 3.73) to C-6 (Figures S7–S11). The analysis conducted above allowed for the determination of the structure of pegaharoline A (Figure 1A).

### 2.2. Pegaharoline A (PA) Inhibited the Proliferation of NSCLC Cells

To determine the influence of PA on NSCLC cell proliferation, A549 and PC9 cells were exposed to various concentrations of PA (ranging from 1.5 to 100 µM) for 48 h. And, the MTT assay was utilized to assess them with paclitaxel as the positive control. The results from the MTT assay demonstrated that PA had potent cytotoxic effects on both A549 and PC9 cells, as evidenced by the IC_50_ values of 2.39 ± 0.27 and 3.60 ± 0.41 μM, respectively (Figure 1B). PA showed stronger antitumor activity against NSCLC cells than those of the plant extract and the positive control paclitaxel (A549: IC_50_ = 4.10 ± 0.12 µM; PC9: IC_50_ = 5.20 ± 0.28 µM) (Table S2). We then used human bronchial epithelial cells (BEAS-2B) to test the toxicity of PA in normal cells and found that the IC_50_ value of PA in BEAS-2B cells was 1.61 ± 0.3 mM (Response Figure 1), which was much higher than its anti-tumor IC50 value (Figure S12). These results indicated that PA was safe in normal cells. Since PA exhibited noteworthy anti-cancer activity, it was subsequently chosen for additional investigation. The accumulation of ROS can lead to apoptosis of cancer cells by overwhelming the cells’ antioxidant defense mechanisms [30]. Multiple drugs with cancer-targeting capabilities have been shown to induce death by elevating ROS levels [31,32]. To determine if PA encourages NSCLC cells by heightening ROS levels, DCFH-DA ROS assay and flow cytometry were carried out. Our results demonstrated that PA, in a dose-dependent manner, increased ROS levels in A549 and PC9 cells (Figure 1C,D). This effect was specifically abrogated by NAC (ROS inhibitor), indicating that the increased ROS levels were due to PA treatment. Therefore, these findings suggest that PA may trigger apoptosis in NSCLC cells by stimulating ROS production.

### 2.3. Pegaharoline A (PA) Inhibited the Expression of CD133 to Activate Autophagy through Blocking the PI3K/AKT/mTOR Pathway

**Figure 2:**
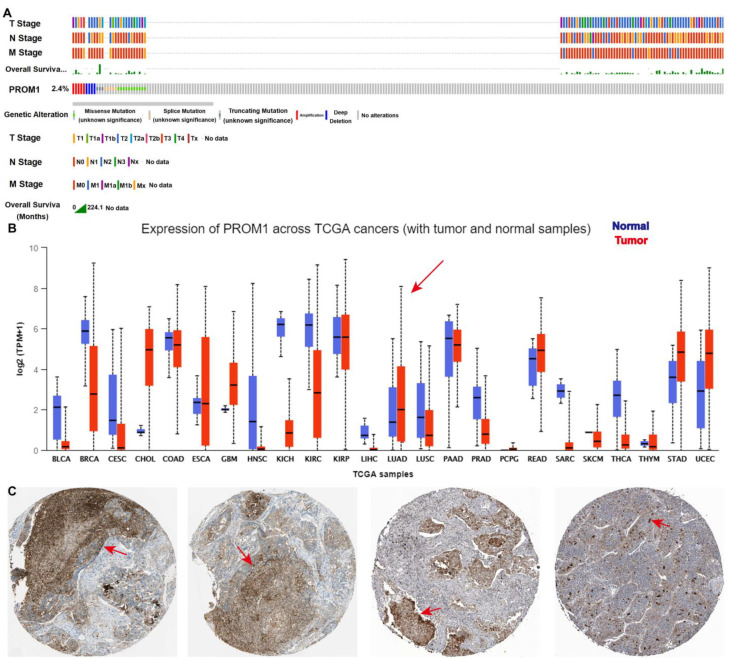
CD133 is overexpressed in lung cancer and strongly correlated with lung cancer “TNM” stage. (**A**) The amplification frequency of CD133 in lung cancer, and CD133 was strongly correlated with lung cancer “TNM” stage. Data were analyzed from cbioportal. (**B**) The expression of CD133 in pan-cancer. The arrows indicates lung adenocarcinoma. Data were analyzed from TCGA. (**C**) CD133 is overexpressed in lung cancer clinical tissue. Data were analyzed from “The Human Protein Atlas”. Scale bar, 100 μm. The arrows indicate overexpressed CD133.

**Figure 3:**
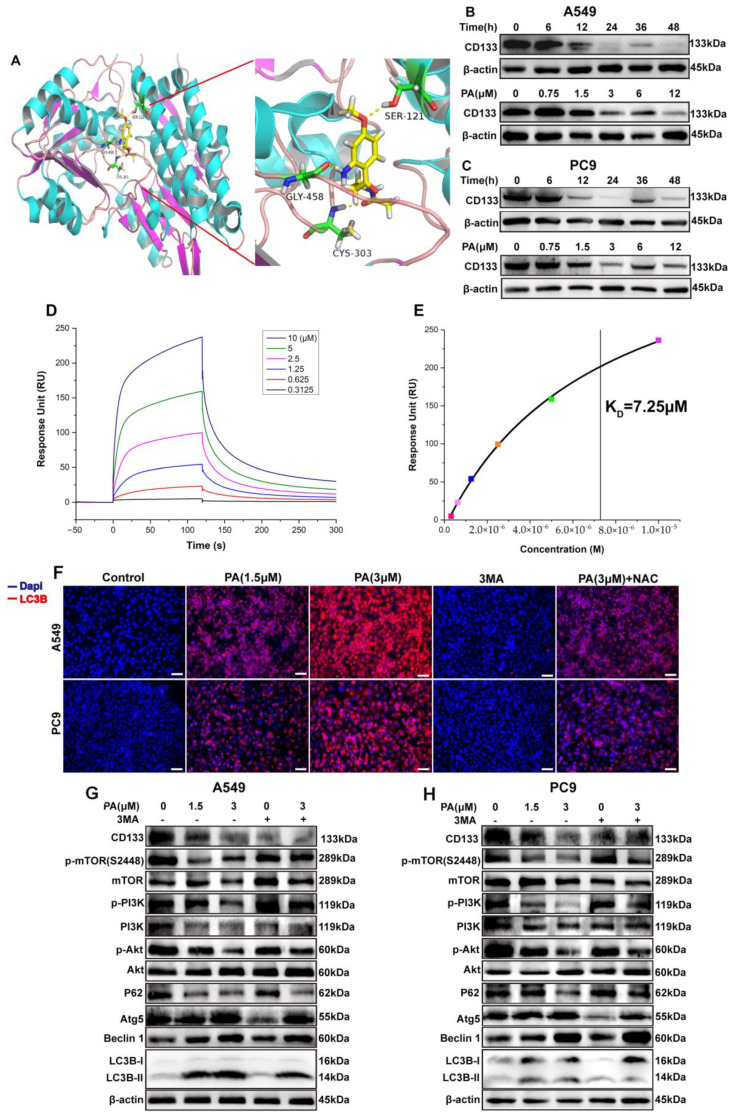
PA (pegaharoline A) inhibits CD133 expression to activate autophagy via blocking the PI3K/AKT/mTOR pathway. (**A**) Molecular docking diagram of ATP binding site between PA and CD133. The A549 and PC9 cells were treated with 3 μM PA at different times or treated for 24 h at different concentrations. The protein levels of CD133 were inhibited by PA both in A549 (**B**) and PC9 (**C**). (**D**) Analysis of the binding affinity of PA for CD133 protein by using SPR. SPR sensing map (**D**) of the interaction of CD133 with various concentrations of PA. The binding affinity curve (**E**) was obtained by fitting the single-site interaction model (different colored squares indicated the response unit (RU) of CD133 with the different concentrations (0.3125, 0.625, 1.25, 2.5, 5.0, 10.0 μM) of PA). (**F**) Immunofluorescence assay examined that treatment with PA for 24 h could promote the expression of autophagic protein LC3B both in A549 and PC9. Scale bar, 100 μm. (**G**,**H**) Western blotting assay detected the protein expression levels of P62, Atg5, Beclin 1, Lc3B, and PI3K/AKT/mTOR-pathway-related protein in A549 (**G**) and PC9 (**H**).

CD133 is widely recognized as a marker for CSCs and is essential in the progression of NSCLC [33]. The bioinformatics analysis from TCGA and “The HUMAN PROTEIN ATLAS” presented that CD133 was overexpressed in NSCLC clinical tissue and strongly correlated with the NSCLC “TNM” stage (Figure 2). To further support this finding, molecular docking studies were performed in order to evaluate the affinity of compound PA for the CD133 active site, with the co-crystallized inhibitor (6-{[(3-fluorophenyl)methyl]sulfanyl}-2-(oxetan-3-yl)-5-phenyl-2,5-dihydro-4*H*-pyrazolo[3,4-d]pyrimidin-4-one) as the reference [34]. The results showed that compound PA fits well in the active site and was held together with the residues SER-121, GLY-458, and CYS-303 by hydrogen bonds inside the binding pocket of CD133 (Figure 3A). The binding score of PA with CD133 was −6.24 kcal/mol (RMSD = 0.42 Å), which was better than that of the co-crystallized inhibitor, with a binding score of −5.71 kcal/mol (RMSD = 0.61 Å). This indicated that PA has more affinity for the CD133 active site than the co-crystallized inhibitor. We conducted a Western blot analysis to determine if PA had any effect on the expression levels of CD133. The results indicated that the CD133 protein levels in A549 and PC9 cells were notably decreased following treatment with PA, as demonstrated in Figure 3B,C. The optimum concentration and time of PA down-regulating CD133 were 24 h and 3 μM, respectively, which were used in further experiments. Furthermore, the binding affinity of PA for the CD133 protein was examined by using the surface plasmon resonance (SPR) method. The response unit (RU) was increased with the increased concentration of PA (Figure 3D). And, the K_D_ value of the interaction of CD133 with PA was 7.25 μM (Figure 3E), which demonstrated that PA had a strong binding affinity for the CD133 protein. CD133 is also associated with autophagy. Hsin et al. discovered that GMI, a fungal immunomodulatory protein, can trigger autophagy to induce the degradation of the CD133 protein. This ultimately induces A549/A400 cell death [35]. To determine the anti-NSCLC mechanism of PA through autophagy, Western blotting, and immunofluorescence assays were carried out. The results (Figure 3F) indicated a significant increase in the fluorescence of LC3B both in A549 and PC9 after PA treatment, which suggested that PA was capable of inducing autophagy in NSCLC cells. Furthermore, the Western blot analysis revealed a dose-dependent reduction in the percentages of p-PI3K/PI3K, p-AKT/AKT, and p-mTOR/mTOR in PA-treated NSCLC cells (Figure 3G,H). Moreover, the protein levels of P62 were remarkably reduced, while Atg5, Beclin 1, and the LC3B-II/LC3B-I ratio were noticeably elevated in PA-treated NSCLC cells. 3MA was an autophagy inhibitor and suppressed autophagy through activating the PI3K/AKT/mTOR pathway and increasing P62 levels while decreasing levels of Atg5, Beclin 1, and a transformation from LC3B-I to LC3B-II. The inhibition of autophagy by 3MA was specially converted by PA (Figure 3F–H). The above data proved that PA could inhibit the expression of CD133 to activate autophagy through blocking the PI3K/AKT/mTOR pathway.

### 2.4. Pegaharoline A (PA) Arrested the Cell Cycle and Promoted Apoptosis of NSCLC Cells

**Figure 4:**
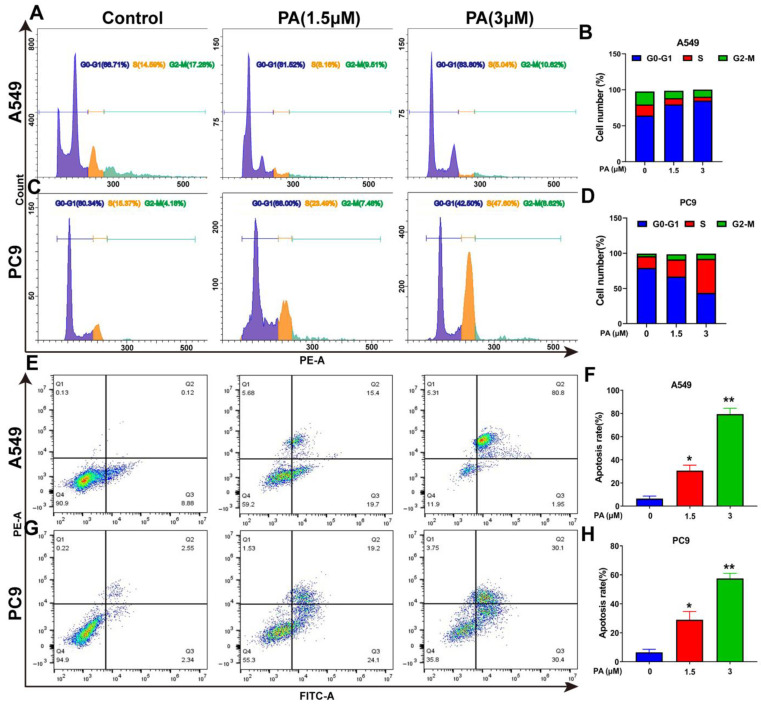

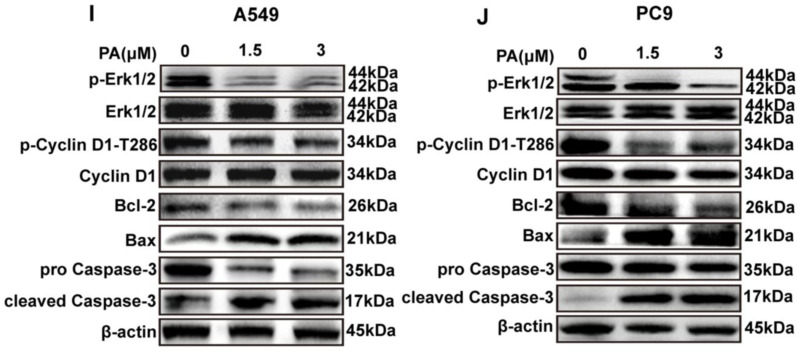
PA inhibits the proliferation of NSCLC cells by arresting cell cycle and promoting apoptosis. (**A**) The effects of PA on A549 cell cycle were detected by flow cytometry. The A549 cells were treated with PA for 24 h. (**B**) Statistical analysis of the A549 cell cycle. (**C**) The effects of PA on PC9 cell cycle were detected by using flow cytometry. The PC9 cells were treated with PA for 24 h. (**D**) Statistical analysis of the PC9 cell cycle. The results are representative of three independent experiments and are expressed as the mean ± SD. (**E**) The effect of PA on A549 cell apoptosis was detected by using flow cytometry. (**F**) Statistical analysis of A549 cell apoptosis. (**G**) The effect of PA on PC9 cell apoptosis was detected by using flow cytometry. (**H**) Statistical analysis of PC9 cell apoptosis. The results are representative of three independent experiments and are expressed as the mean ± SD. * *p* < 0.05, and ** *p* < 0.01 compared with the control group. The effects of PA on cell-cycle-related and apoptosis-related proteins in A549 cells (**I**) and PC9 cells (**J**) were detected by using Western blotting assay.

Following a 24 h treatment with PA, the A549 cell ratio of G0-G1 phases increased from 66.71% to 83.80%, while the portion of S and G2-M phases decreased from 14.59% and 17.28% to 5.04% and 6.71%, respectively (Figure 4A,B). In addition, the PC9 cell ratio of S and G2-M phases increased significantly, with the ratio rising from 15.37% and 4.18% to 47.60% and 8.62%, respectively, while the portion of G0-G1 decreased from 80.34% to 42.50% after treatment with PA (Figure 4C,D). The molecular mechanism of the cell cycle arrest was evaluated via Western blotting analysis. In both A549 and PC9 cells, the p-Cyclin D1/Cyclin D1 and p-Erk1/2/Erk1/2 ratios were decreased in the PA-treated group (Figure 4I,J). Flow cytometry analysis was utilized to detect the PA-treated cells, revealing a notable dose-dependent increase in the apoptosis rate of both A549 and PC9 cells (Figure 4E–H). To explore the molecular evidence of apoptosis, apoptosis-related protein levels were determined [36]. The group treated with PA exhibited a decrease in Bcl-2 protein levels, along with an increase in Bax and cleaved caspase-3 levels, ultimately enhancing the apoptosis of A549 and PC9 cells (Figure 4I,J).

### 2.5. Pegaharoline A (PA) Inhibits DNA Synthesis, Clone Formation, and Angiogenesis of HUVECs

**Figure 5:**
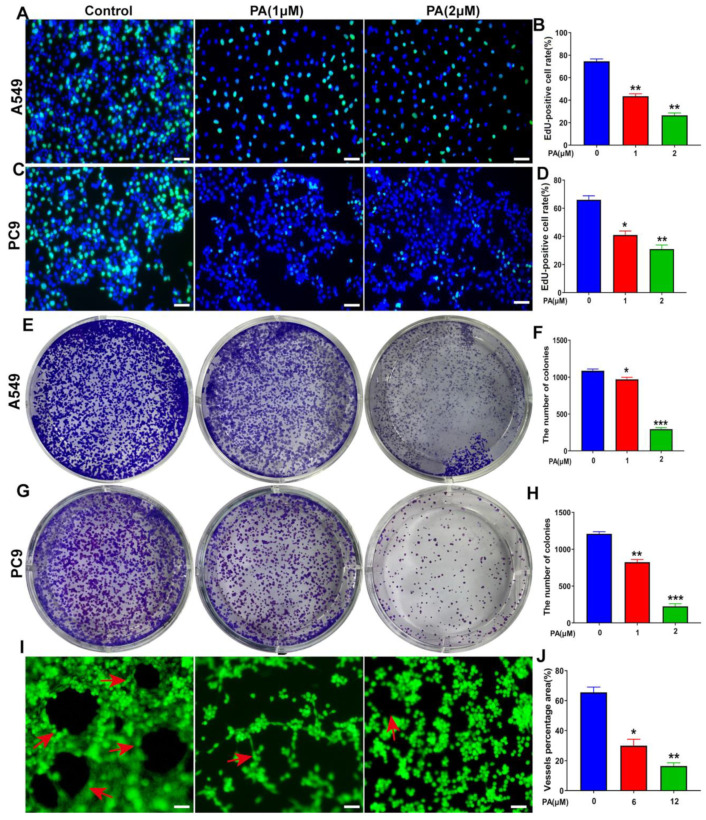
PA inhibited DNA synthesis, clone formation, and angiogenesis of HUVECs. (**A**) The effect of PA on DNA synthesis in A549 cells was detected by using EdU assay. The A549 cells were treated with PA for 24 h. Scale bar, 100 μm. (**B**) Statistical analysis of EdU-positive cells in A549 cells. (**C**) The effect of PA on DNA synthesis in PC9 cells was detected by using EdU assay. The PC9 cells were treated with PA for 24 h. Scale bar, 100 μm. (**D**) Statistical analysis of EdU-positive cells in PC9 cells. (**E**) The effect of PA on the colony formation of A549 cells was detected by colony formation assay. (**F**) Statistical analysis of the clone number of A549 cells after treatment with PA. (**G**) The effect of PA on the colony formation of PC9 cells. (**H**) Statistical analysis of the clone number of PC9 cells after treatment with PA. (**I**) The effect of PA on angiogenesis in HUVECs. Arrows: the angiogenesis-like structure. Scale bar, 100 μm. (**J**) The area ratio of vascular formation in HUVEC after treatment with PA. The results are representative of three independent experiments and are expressed as the mean ± SD. * *p* < 0.05, ** *p* < 0.01, and *** *p* < 0.001 compared with the control group.

The influence of PA on DNA synthesis was analyzed through the use of the EdU staining method in NSCLC cells. The fluorescence intensity of Edu was observed to have a notable reduction in both A549 and PC9 cells in the PA-treated group (Figure 5A–D). This outcome suggests that PA has the ability to impede DNA synthesis in NSCLC cells. Moreover, PA was found to significantly decrease the clone formation of NSCLC cells (Figure 5E–H). The formation of tubes in HUVECs was assessed, and the results revealed a significant decrease in tube formation within the PA-treated group, as depicted in Figure 5I,J. The MTT results from Response Figure 1 showed that the IC_50_ value of PA in HUVECs was 0.28 ± 0.2 mM (Figure S12), which indicated that PA-inhibited angiogenesis of HUVECs does not depend on its inhibition effects. Finally, these data demonstrated that PA could suppress the proliferation of NSCLC cells through inhibiting the DNA synthesis, clone formation, and angiogenesis of HUVECs.

### 2.6. Pegaharoline A (PA) Inhibited the Invasion and Migration Abilities of NSCLC Cells through Inhibiting the EMT Signal Pathway

**Figure 6:**
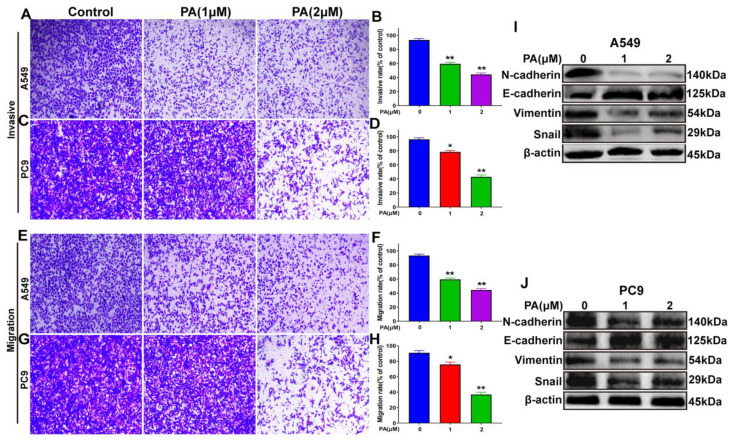
PA inhibited the invasion and migration abilities of NSCLC cells by blocking EMT signal pathway. (**A**) The effects of PA on the invasion of A549 cells. Scale bar, 100 μm. (**B**) Statistical analysis of A549 cell invasive ability after treatment with PA. (**C**) The effects of PA on the invasion of PC9 cells. Scale bar, 100 μm. (**D**) Statistical analysis of PC9 cell invasive ability after treatment with PA. (**E**) The effects of PA on the migration of PC9 cells. Scale bar, 100 μm. (**F**) Statistical analysis of PC9 cell migration ability after treatment with PA. (**G**) The effects of PA on the migration of PC9 cells. Scale bar, 100 μm. (**H**) Statistical analysis of PC9 cell migration ability after treatment with PA. The effect of PA on the expression of EMT-related proteins in A549 (**I**) and PC9 (**J**) was detected by using Western blotting assay. Compared with the control group, the PA-treated group inhibited the EMT signal pathway by decreasing the expression of N-cadherin, Vimentin, Snail, while increasing the expression of E-cadherin. The results are representative of three independent experiments and are expressed as the mean ± SD. * *p* < 0.05 and ** *p* < 0.01 compared with the control group.

Analysis of NSCLC cells using microscopy imaging and quantitative methods showed that the NSCLC cells treated with PA exhibited slower invasion rates than those that were untreated (Figure 6A–D). Moreover, the migratory capability of PA-treated NSCLC cells was suppressed (Figure 6E–H). To verify the mechanisms of PA in inhibiting the invasion and migration abilities of A549 and PC9 cells, a Western blot analysis was performed. The results (Figure 6I,J) showed that N-cadherin, Vimentin, and Snail protein levels were obviously decreased in PA-treated group, and E-cadherin protein levels were increased. This demonstrated that PA suppressed the invasive and migratory capabilities through inhibiting the EMT signaling pathway.

## 3. Discussion

As the current medications for NSCLC have numerous adverse effects, there is a need to discover new alternative molecules for effective clinical treatment. In this work, the examination of the P. harmala alkaloids resulted in the isolation of a new tryptamine-derived alkaloid (PA), which was identified through various spectroscopic data. In addition, the cytotoxicity of PA was found to be high on A549 and PC9 cells with IC_50_ values of 2.39 ± 0.27 and 3.60 ± 0.41 μM, respectively. Further investigation was conducted to explore the cytotoxic mechanism of PA, which revealed that it caused a dose-dependent inhibition of cell proliferation and blocked the cell cycle. The cell cycle results in its duplication and division in a cyclical manner, including activities of cell proliferation and differentiation [37]. One strategy to hinder the growth of cancerous cells is to interfere with specific checkpoints during the cell cycle. Various intracellular signaling pathways can be targeted by anticancer drugs to inhibit proliferation by interrupting the cellular processes at different stages. The Ras-Raf-MEK-ERK MAP pathway is critical for various processes, such as cell proliferation, apoptosis, and RNA synthesis and processing, and is mediated by key protein kinases ERK1 and ERK2 [38]. Cyclin D1 is important in cellular progression by regulating the activity of CDK and CDK6 during the conversion of the G1 to S phase [39]. To confirm the arrest at the cell cycle, the Erk1/2 and Cyclin D1 protein levels were measured. The reduction in phosphorylation levels of Erk1/2 and Cyclin D1 observed in PA-treated cells was in agreement with the results of flow cytometry analysis. These results indicated that PA arrested cellular progression at G0/G1 stage to inhibit cell proliferation through suppressing the activation of Erk1/2 and Cyclin D1.

Apoptosis is a natural process in which cells intentionally self-destruct, and it is an important autoregulated mechanism to maintain a healthy environment within multicellular organisms. The involvement of the mitochondria-mediated pathway is crucial for caspase-dependent apoptosis to occur. Following the activation of an apoptosis-inducing signal, Bcl-2 apoptosis protein levels are regulated. Then, cytochrome c is induced to be released from the mitochondria, which could trigger apoptosis through the activation of the caspase cascade. Caspase-3 is an important protease enzyme in the apoptosis execution stage, and its activation is initiated by the cytochrome c apoptosome [40]. The expression levels of marker proteins of apoptosis were analyzed to assess the apoptotic cell death of PA-treated NSCLC cells. The results demonstrated that, in PA-treated groups, Bcl-2 protein levels were reduced, while Bax and cleaved caspase-3 levels were enhanced. This suggests that the caspase-3 pathway is activated, ultimately leading to apoptosis induction. In our study, the level of caspase 8 was not detected by Western blotting, which indicates that PA induced NSCLC cell apoptosis possibly through the intrinsic apoptosis pathway.

Autophagy is significant for maintaining cellular homeostasis, immune response, and development. It is a genetically regulated, self-destructive behavior that has been a possible focus for cancer therapy. LC3B and P62 are marker proteins for autophagy. During autophagy, the LC3BI protein is converted to LC3BII and is subsequently localized to the membrane of the autophagosome. This is considered to be one of the markers of autophagy induction in cells. P62/SQSTM1 can bind with LC3 through a short LC3 interaction region (LIR) and then be specifically degraded by autophagy. The level of p62 expression is inversely related to autophagic activity, and it can be used as a supplementary marker to monitor autophagy [41]. Our data indicated that PA has a significant effect on inducing autophagy in NSCLC cells, as evidenced by the increased LC3BII levels and decreased P62 levels. The mTOR/PI3K/AKT pathway is an important regulatory route for controlling cell proliferation and promoting the tumorigenesis of various cancers. Because of nutrient deficiency, LBK/AMPK is triggered to prevent the interaction between mTORC1 and ULK, thereby inhibiting the activity of mTOR. ULK1 then phosphorylates ATG13 and FIP200 to form autophagosomes and, ultimately, induce autophagy [42]. In this study, PA blocked the mTOR/PI3K/AKT pathway by inhibiting the phosphorylation of key signaling molecules involved in this pathway, such as mTOR, PI3K, and AKT, to activate autophagy. Autophagy could potentially regulate the expression of CD133 by modulating the population of CSCs [43]. Our findings demonstrated that treatment with PA could effectively diminish the CD133 protein levels of NSCLC cells. Interestingly, the autophagy inhibitor 3MA could also decrease levels of CD133. The homeostasis of autophagy was important for modulating CD133 expression. Additional experiments are necessary to clarify the mechanism of CD133 regulation by autophagy.

EMT is a significant factor in cellular transformations, resulting in detachment from neighboring cells, loss of apicobasal polarity, increased cellular elongation, and greater mobility. Furthermore, this transformation represents a crucial element in the initiation of the metastasis cascade, which is essential for cancer progression [44]. The EMT process results in a reduction in the E-cadherin level and an increase in N-cadherin, vimentin, and snail levels [45]. This study showed a significant reduction in the invasive and migratory capabilities of PA-treated NSCLC cells. In addition, Western blotting showed that N-cadherin, vimentin, and snail levels were decreased following PA treatment, while E-cadherin levels were up-regulated. Overall, the findings suggested that PA had the potential to hinder the invasive and migratory capabilities of NSCLC cells by blocking the EMT pathway.

## 4. Materials and Methods

### 4.1. Experimental Instruments and Chemicals

NMR, UV, and IR spectra were obtained using Bruker AV-600 (Bruker; Ettlingen, Germany), JASCO V-550 UV-VIS (Jasco; Tokyo, Japan), and JASCO FT/IR-480 plus FT-IR spectrometers (Jasco; Tokyo, Japan), respectively. LC-QTOF-MS data were generated using a Shimadzu LC-20AD liquid chromatogram (Shimadzu; Kyoto, Japan) and an AB SCIEX X500R QTOF mass spectrometer (AB SCIEX; Framingham, MA, USA). The MTT reagent was obtained from Keygen Biotech located in Nanjing, China. The PI reagent was obtained from Sigma-Aldrich^®^, (St. Louis, MO, USA). The RNase A reagent was procured from Fermentas^®^ (Shanghai, China). Detailed instruments and chemicals are provided in the Supporting Information.

### 4.2. Plant Material

In July 2017, the *P. harmala* L. seeds were sourced from Xinjiang Province, China, and were recognized by Professor Guangxiong Zhou (Jinan University). The sample voucher (20170716) was saved in the School of Pharmacy at Jinan University, China.

### 4.3. Extraction and Isolation

A total of 12 kg of powdered *P. harmala* seeds were subjected to extraction using 30 L of 95% ethanol three times. After concentrating the extract (956 g), it was combined with 4 L water, and the pH level was reduced to 3 through the addition of 5% HCl. The mixture was then extracted three times with CH_2_Cl_2_ (4 L each time) to obtain a fraction. The acid phase was subsequently adjusted to pH 9 using 30% NH_3_∙H_2_O. Then, the alkali layer was successively partitioned with CH_2_Cl_2_ (4 L × 3) to yield the total alkaloid extract (315.0 g). To purify the crude alkaloids, D101 macroporous adsorption resin column chromatography was employed with EtOH/H_2_O (20:80, 40:60, 60:40, 80:20, 100:0, *v*:*v*), which resulted in the separation of five fractions (Fr. A–E). Fraction C (22.3 g) was employed on silica gel CC with a system of CH_2_Cl_2_/MeOH (10:90 to 100:0, *v*/*v*), resulting in the separation of seven subfractions (Fr. C1–C7). Sephadex LH-20 was used for the purification of Fr. C5 (1.8 g), resulting in the isolation of compound PA (22.1 mg).

Pegaharoline A (PA): ${\left[\alpha \right]}_{D}^{25}$ + 13.7 (c 1.0, CH_2_Cl_2_); White solid; IR (KBr) *ν*_max_ 3406, 3329, 3178, 3048, 1684, 1616, 1468, 885, 775, 683 cm^−1^; UV (CH_2_Cl_2_) λ_max_ 205, 234, 287 nm; Please refer to Table S1 for the NMR spectroscopic data; HR-ESI-MS data: *m*/*z* 237.1233 (calcd. for C_12_H_17_N_2_O_3_, [M + H]^+^, 237.1234).

### 4.4. Cell Culture

A549 and PC9 cells were kindly provided by Sun Yat-Sen University, which were grown in RPMI-1640 medium containing 10% FBS and preserved at a temperature of 37 °C with 5% CO_2_ for a duration of 24 h.

### 4.5. MTT Assay

When A549 and PC9 cells were at the logarithmic phase, they were prepared as cell suspensions using a culture medium containing 10% FBS, and 5 × 10^3^ cells were seeded into a 96-well plate with a volume of 200 μL per well. Following a 24 h incubation period, different concentrations of compound PA were added to A549 and PC9 cells. They were subsequently cultivated at 37 °C with 5% CO_2_ for 48 h. Then, 20 μL of MTT (5 mg/mL) was added to each well. After incubating for an additional 4 h, the culture was stopped, and the supernatant was carefully aspirated from each well. An amount of 150 μL of DMSO was added to each well, and shaken gently for 10 min to fully dissolve the crystals. The absorbance value of each well was measured at a wavelength of 490 nm using an ELISA reader.

### 4.6. Measurement of ROS

ROS levels were evaluated using the ROS assay with DCFH-DA. When A549 and PC9 cells were at the logarithmic phase, they were inoculated at a density of 4 × 10^5^ cells/mL onto a 6-well plate and exposed to PBS for a period of 24 h. Following rinsing with PBS, a solution of 10 μM DCFH-DA was used to stain A549 and PC9 cells for 30 min in the absence of light. A fluorescence microscope and flow cytometer were utilized to determine the levels of ROS.

### 4.7. Molecular Docking Studies

The Surflex-Dock tool was utilized to conduct molecular docking studies in Tripos SYBYL 8.0 software [46,47,48]. The crystal structure of CD133 protein (PDB ID: 6DUM) was obtained from the RCSB Protein Data Bank (https://www.rcsb.org/, accessed on 1 July 2021). The protein structure of CD133 was treated by removing water molecules and performing hydrogenation. And, the structural optimization on PA was performed through hydrogenation and energy minimizing. Tripos SYBYL was used for molecular docking operations and to visualize and analyze the results.

### 4.8. Immunofluorescence Assay

The expression of LC3B was examined through an immunofluorescence test. NSCLC cells that underwent various treatments were immobilized using frozen pure methanol for a duration of 20 min at −20 °C. Once treated with non-fat milk, the NSCLC cells under various groups were exposed to primary antibodies and kept at a temperature of 37 °C for a period of 2 h. Subsequently, the NSCLC cells were rinsed with TBST and marked with suitable secondary antibodies (Abcam; ab150080; Cambridge; UK) that were diluted at 1:500 for 1 h at a temperature of 37 °C. Next, a solution of DAPI was used to stain NSCLC cell nuclei for 20 min. Finally, a fluorescence microscope was used to acquire photographs of the stained cells.

### 4.9. Western Blotting

A549 and PC9 cells, which were at logarithmic phase and good growth status, were placed in a six-well plate at 2 × 10^5^ cells/well and grown for 24 h before being treated with the compound PA. Next, RIPA solution was applied and then the NSCLC cells were lysed for 10 min. A BCA technique was employed to determine the protein density of the above-collected NSCLC cells. According to the result, the protein density and volume in all samples were adjusted to be the same. The same quantity of protein was applied to every track for the SDS-PAGE gels. The activating PVDF membrane, which was soaked in methanol for 3–5 min, were used to transfer the SDS-PAGE gels. After transferring, the PVDF membrane was removed, and the methanol on the residual membrane was cleaned with ultra-pure water. The membrane was sealed with 5% non-fat milk for more than 1 h. After the closure, the membranes were washed with PBST on a shaker 3 times, for 10 min each. Thus, primary and secondary antibodies were loaded onto the PVDF membranes to incubate at low speed on a shaker at 4 °C for 12 and 2 h, respectively. Finally, electrochemiluminescence solutions were dripped onto the incubated PVDF membrane, and the visualized Western blot data were recorded using the Azure Biosystems C500 system (Azure Biosystems; Dublin, CA, USA).

### 4.10. Apoptosis Assay

A549 and PC9 cells were placed in six-well plates, with 2 × 10^5^ cells/well, and incubated for a period of 24 h. Then, cells were exposed to specific concentrations of the compound PA. After an additional 24 h incubation period, the cells were detached using trypsin and then centrifuged at 2000 rpm for 5 min. Finally, according to the instructions of a Cell Apoptosis Detection Kit provided by the manufacturer, 0.1 mL Annexin V binding buffer containing 1 μg/mL PI was added to stain NSCLC cells for 15 min. The acquisition and analysis of data were carried out by means of FACS Calibur.

### 4.11. Cell Cycle Assay

A549 and PC9 cells that had been synchronized were washed twice with PBS. Cooled 75% ethanol was added to culture with cells at 4 °C for 6 h. After that, a solution of PI (10 μg/mL) with addition of RNase A was used to stain NSCLC cells for 30 min in the absence of light. Finally, flow cytometry was employed to acquire the data on the cell cycle.

### 4.12. EdU Staining

A549 and PC9 cells were placed in six-well plates, with 1.5 × 10^5^ cells/well, and incubated for a period of 24 h. Then, cells were exposed to specific concentrations of the compound PA. After an additional 48 h incubation period, 0.5 mL reaction cocktail of Click-iT Plus was added to every well to cover the cells completely. The cells were left to incubate in the dark for 30 min. Next, a solution of DAPI was used to stain NSCLC cell nuclei for 10 min in dark. Finally, a fluorescence microscope was used to acquire photographs of the stained cells.

### 4.13. Colony Formation Assay

A549 and PC9 cells were placed in six-well plates, containing a density of 1 × 10^3^ cells/well, and incubated for a period of 24 h. Subsequently, NSCLC cells were subjected to PA treatment at concentrations of 0, 12.5, 25.0, and 50.0 μM for a duration of 48 h. After the replacement of medium, and the cells were left to incubate for an additional 15 days. After the incubation period, a solution of 4% paraformaldehyde was used to fix NSCLC cells for 30 min. Next, a solution of 0.1% crystal violet was applied to NSCLC cells to stain for 20 min. Finally, colonies were quantified and recorded by a camera.

### 4.14. Transwell Test

To perform the invasion experiment, an artificial basement membrane called Matrigel was applied to the upper surface of the transwells and incubated for 12 h. NSCLC cells (with a concentration of 1 × 10^5^ cells) were subjected to different treatments and then cultured on the Matrigel within the upper chambers of the transwell, using a medium containing 1% FBS. Concurrently, a medium with 20% FBS was introduced into the lower side of the Transwell. After a period of 24 h, NSCLC cells were removed from the upper side of the membrane. Following their migration to the opposite side of the filter, a solution of 4% paraformaldehyde was used to fix NSCLC cells for 20 min. Next, a solution of 0.5% crystal violet was applied to NSCLC cells to stain for 20 min. The number of the stained cells was determined by capturing images of three randomly selected fields using a microscope, with a magnification of ×200.

### 4.15. Angiogenesis of HUVEC Assay

The Matrigel matrix glue was stored in a refrigerator at a temperature of 4 °C for 24 h. Next, Matrigel was placed in 96-well plates and allowed to solidify at a temperature of 37 °C for a duration of 1 h. Next, a quantity of 1.5 × 10^4^ HUVECs was placed in each well containing the Matrigel bed and was left to incubate for 6 h. Image Pro^®^ Plus 6.0 software was applied to the image and quantified the tube formation and lengths.

### 4.16. Statistical Analysis

The results were replicated at least three times, which were presented as the means with standard deviation (S.D.) to provide a measure of the variation within the results. The results were analyzed using SPSS version 25.0 software.

## 5. Conclusions

**Figure 7:**
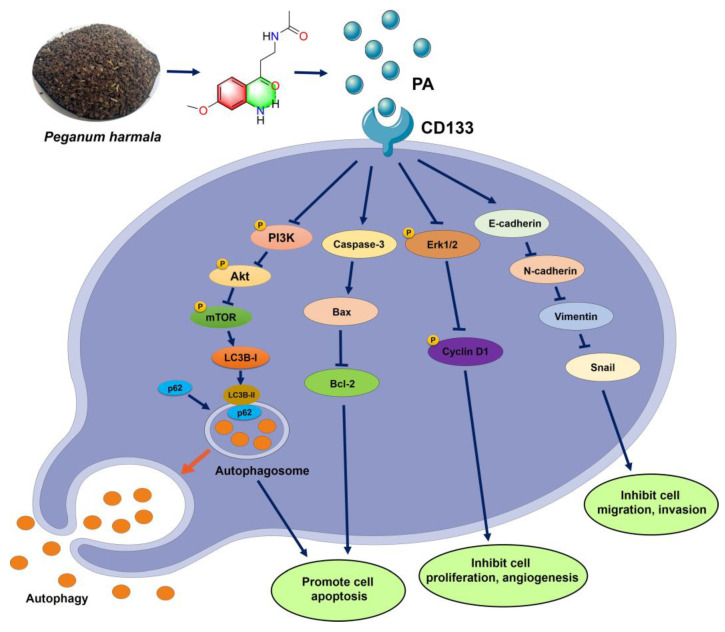
Mechanism schematic diagram of PA suppressing NSLC cells.

This study reported the isolation of a novel aniline derivative (PA) from *P. harmala* seeds and its related anti-tumor mechanism in NSCLC. The structure of PA was identified through spectroscopic techniques, such as HR-ESI-MS, IR, UV, 1D-, and 2D-NMR. The MTT, colony formation, EdU staining, transwell assay, etc., all demonstrated that PA could inhibit NSCLC cell growth, migration, and invasion. At the molecular level, the docking data and SPR revealed that PA could bind with CD133 to decrease its expression, resulting in activating autophagy of NSCLC cells by inhibiting the PI3K/AKT/mTOR pathway. Meanwhile, PA increased ROS levels, Bax, and cleaved caspase-3 to promote apoptosis and inhibited cell migration and invasion through blocking the EMT pathway. This finding confirms PA as a potential anticancer therapeutic in NSCLC cells through targeting CD133 to block the PI3K/AKT/mTOR and EMT pathways (Figure 7) [49,50,51]. Additional research is required to detect whether PA could combine with other clinical drugs to improve NSCLC therapy.

## Data Availability

The data presented in this study are available upon request from the corresponding author.

## Supplementary Materials

The supporting information can be downloaded at: https://www.mdpi.com/article/10.3390/ijms241612626/s1.
