# Supplementary material for: A Novel Aniline Derivative from Peganum harmala L. Promoted Apoptosis via Activating PI3K/AKT/mTOR-Mediated Autophagy in Non-Small Cell Lung Cancer Cells
Source: Int J Mol Sci. 2023 Aug 10;24(16):12626. doi: 10.3390/ijms241612626 (PMC10454575; doi:10.3390/ijms241612626)
Supplement: Supplementary file 1 [file ijms-24-12626-s001.zip › ijms-2490460-SI.pdf]

Supporting Information

Contents: **page**

General Experimental Procedures..1
Biogenetic relationships of pegaharoline A (PA) 2
Scheme S1. Proposed biosynthetic pathways for PA..2
Table S1. NMR data of PA (DMSO-d_6, 600 MHz for ^{1}H, 150 MHz for ^{13}C, δ in ppm, J in Hz).....3
Table S2 Cytotoxic activities of PA, total alkaloids extract, and the paclitaxel on NSCLC cells.....3
Figure S1. HR-ESI-MS of PA..4
Figure S2. UV spectrum of PA (CH_2Cl_2) 4
Figure S3. IR spectrum of PA (KBr disc)..5
Figure S4. ^{1}H NMR spectrum of PA in DMSO-d_6 (600 MHz) 5
Figure S5. ^{13}C NMR spectrum of PA in DMSO-d_6 (150 MHz)......................................6
Figure S6. DEPT-135 spectrum of PA in DMSO-d_6 (150 MHz)..6
Figure S7. Key ^{1}H-^{1}H COSY and HMBC correlations of PA. 7
Figure S8. ^{1}H-^{1}H COSY spectrum of PA in DMSO-d_6 7
Figure S9. HSQC spectrum of PA in DMSO-d_6...7
Figure S10. HMBC spectrum of PA in DMSO-d_6...8
Figure S11. NOESY spectrum of PA in DMSO-d_6..8
Figure S12. The toxicity of PA in BEAS-2B and HUVECs...9

General Experimental Procedures

Optical rotations were measured using a JASCO P-1020 polarimeter (Jasco; Japan). UV spectra were obtained on a JASCO V-550 UV-VIS spectrophotometer (Jasco; Japan). IR spectra of all the compounds were obtained by recording the absorption of thin films with KBr pellets using a JASCO FT/IR-480 plus FT-IR spectrometer (Jasco; Japan). NMR spectra were obtained using a Bruker AV-600 spectrometer (Bruker; Germany) operating at 600 MHz for ^{1}H and 125 MHz for ^{13}C. HR-ESI-MS data were acquired using an Agilent 6210 ESI/TOF mass spectrometer (Agilent; USA). LC-QTOF-MS data were generated using a Shimadzu LC-20AD liquid chromatogram (Shimadzu; Japan) and an AB SCIEX X500R QTOF high resolution hybrid quadrupole time-of-flight mass spectrometer (AB SCIEX; USA).

The MTT reagent was obtained from Keygen Biotech located in Nanjing, China. The RPMI-1640 medium, FBS, PBS, sodium pyruvate, trypsin and antibiotics were procured from HyClone (GE Healthcare Life Sciences, USA). The PI reagent was obtained from SigmaAldrich®, (St. Louis, USA). The RNase A reagent was procured from Fermentas® (Shanghai, China). The Click-iT Plus EdU Alexa Fluor 594 Imaging Kit, Hoechst 33342 and Phosphate-buffered saline with 0.05% Tween-20 (PBST) were obtained from Thermo Fisher Scientific (Waltham, MA, USA). The Matrigel matrix glue was procured from BD Biosciences (San Diego, USA). CellTiter 96® AQueous One Solution Cell Proliferation Assay kit was obtained from Promega Corp. (Madison, USA). The Annexin V/PI Cell Apoptosis Detection Kit was acquired from KeyGen Biotech (Jiangsu, China). The PVDF membrane was procured from EMD Millipore (Billerica, MA, USA). The molecular weight markers were acquired from Fermentas (Thermo Fisher Scientific, USA). The BCA protein assay kit and RIPA buffer were sourced from Beyotime Institute of Biotechnology (Shanghai, China). The freeze-dried CDDP powder was bought from Qilu Pharmaceutical Co., Ltd. (Jinan, China). The anti-Bcl-2, anti-Bax, anti-caspase 3, anti-LC3B, anti-p-Akt (Ser473), anti-Akt 1/2/3, anti-p-mTOR (Ser2448), anti-mTOR, anti-p-PI3K (Try 458), and anti-PI3K antibodies were obtained from Cell Signalling Technology (Beverly, MA, USA).

Biogenetic relationships of pegaharoline A (PA)

Proposed biosynthetic pathways for pegaharoline A are shown in **Scheme S1**. Pegaharoline A could originate from tryptamine. First, tryptamine could be oxidized to give peganumaline B [1]. Then, the C-N bond of the pyrrole ring in peganumaline B was cleaved to afford the intermediate **A1**. Finally, pegaharoline A could be formed by the degradation of the aldehyde group and then oxidation of the oxygenated quaternary carbon of **A1**.

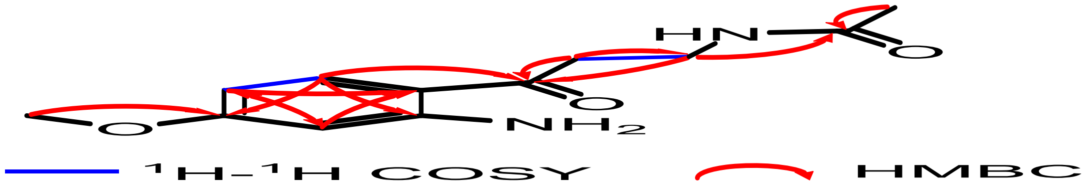

Scheme S1. Proposed biosynthetic pathways for pegaharoline A.

References

[1] K.B. Wang, X. Hu, S.G. Li, X.Y. Li, D.H. Li, J. Bai, Y.H. Pei, Z.L. Li, H.M. Hua, Racemic indole alkaloids from the seeds of *Peganum harmala*, Fitoterapia 125 (2018) 155–160.

Table S1. NMR data of PA (DMSO-d_6, 600 MHz for ^{1}H, 150 MHz for ^{13}C, δ in ppm, J in Hz).

Position	δ_H	δ_C	^{1}H-^{1}H COSY	HMBC
2	-	153.6	-	-
3	-	111.3	-	-
4	7.64 (1H, d, 9.0)	133.3	H-4	C-1,5,7
5	6.14 (1H, dd, 9.0, 2.4)	103.6	H-3	C-2,6
6	-	163.8	-	-
7	6.24 (1H, d, 2.4)	98.5	-	C-2,4
8	-	198.4	-	-
9	2.98 (2H, t, 6.8)	38.1	H-9	C-7,9
10	3.32 (2H, t, 6.8)	34.9	H-8	C-7,8,11
12	-	169.3	-	-
13	1.77 (3H, s)	22.6	-	C-11
6-OCH_3	3.73 (3H, s)	55.0	-	C-5
NH_2-1	7.34 (2H, br s)	-	-	-
NH-11	7.89 (1H, t, 5.0)	-	-	C-9,11

Table S2. Cytotoxic activities of PA, total alkaloids extract, and the paclitaxel on NSCLC cells.

Compounds	IC_{50}[a]	
	**A549**	**PC9**
PA	2.39 ± 0.27 μM (μmol/L)	3.60 ± 0.41 μM
Total alkaloids extract	18.92 ± 0.60 μg/mL	21.20 ± 0.90 μg/mL
Paclitaxel[b]	4.10 ± 0.12 μM	5.20 ± 0.28 μM

[a] IC_{50} values were detected by MTT assay after incubation for 48 h; data are expressed as mean ± SD;

[b] Positive control.

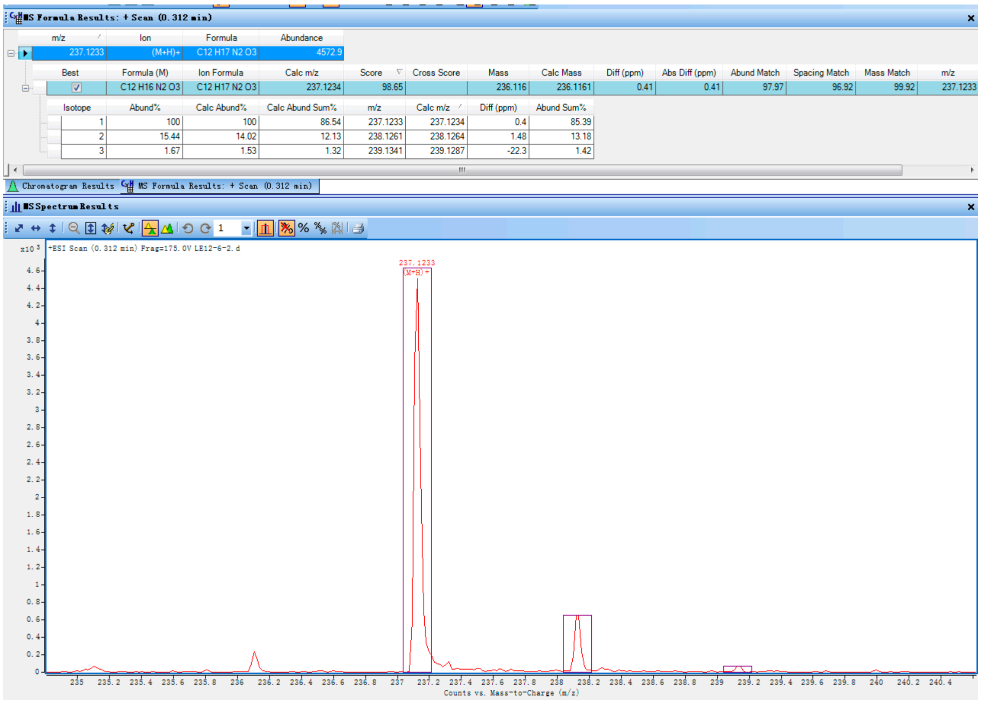

Figure S1. HR-ESI-MS of PA.

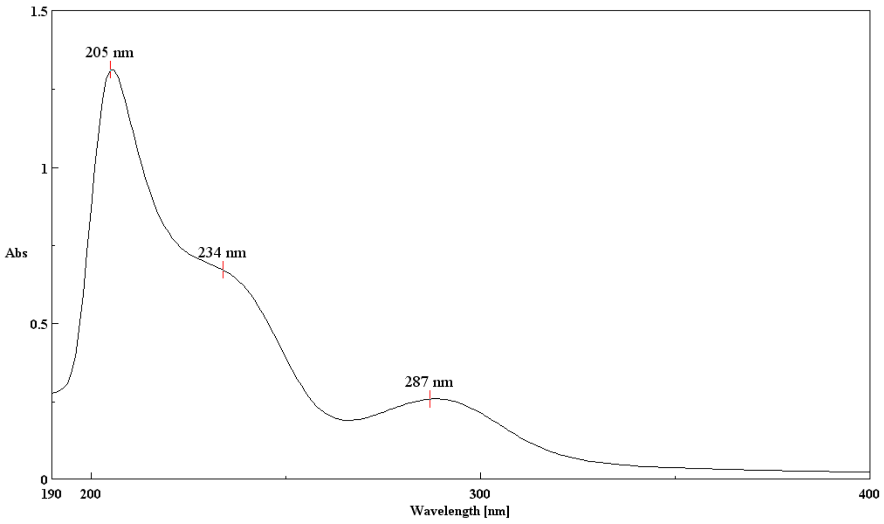

Figure S2. UV spectrum of PA (CH_2Cl_2).

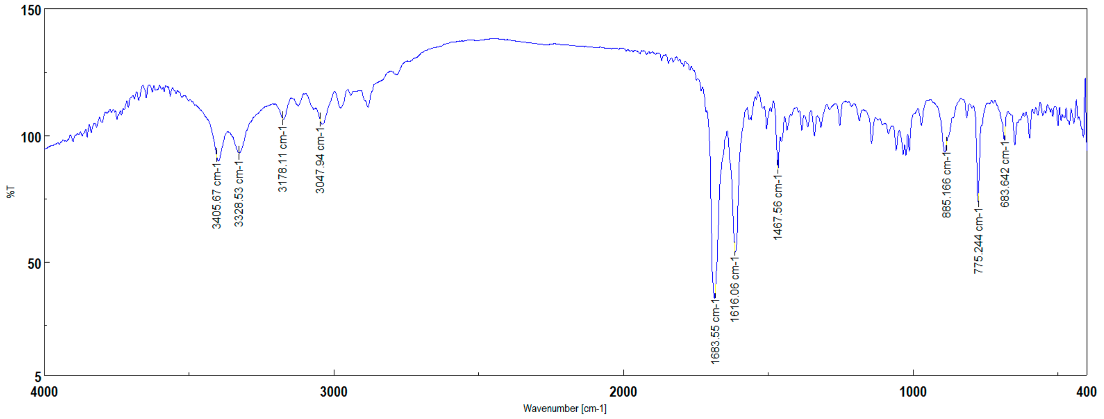

Figure S3. IR spectrum of PA (KBr disc).

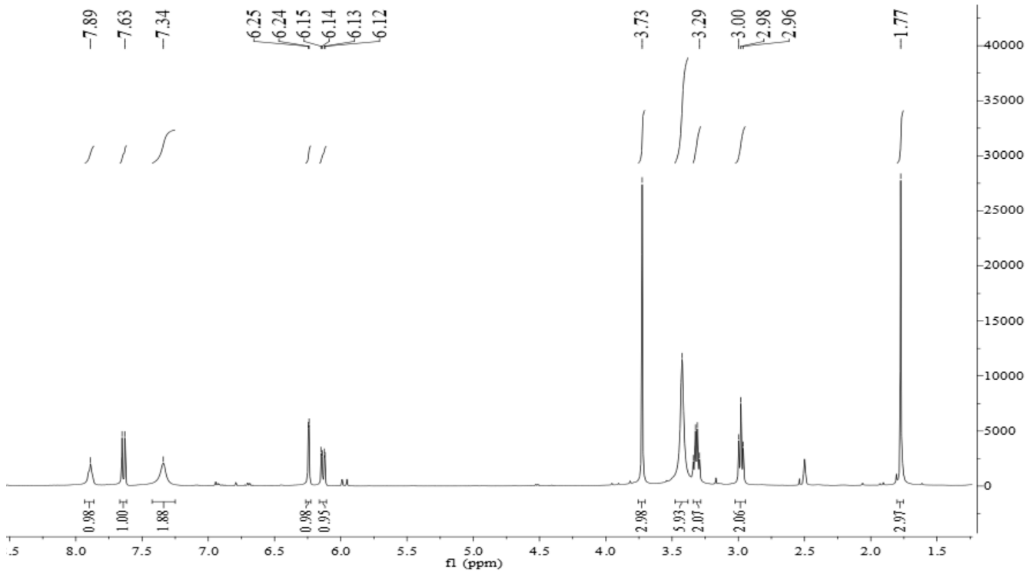

Figure S4. ^{1}H NMR spectrum of **PA** in DMSO-d_6 (600 MHz).

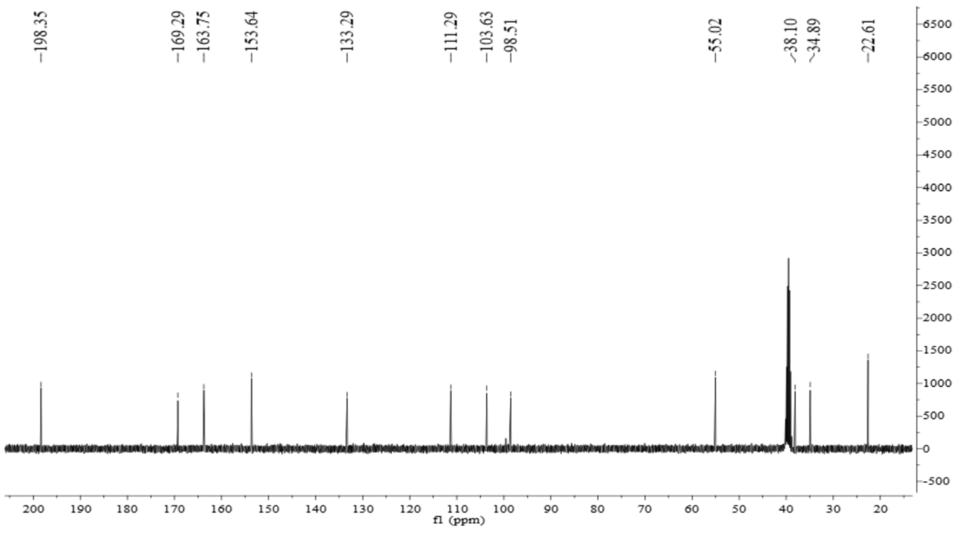

Figure S5. ^{13}C NMR spectrum of PA in DMSO-d_6 (150 MHz).

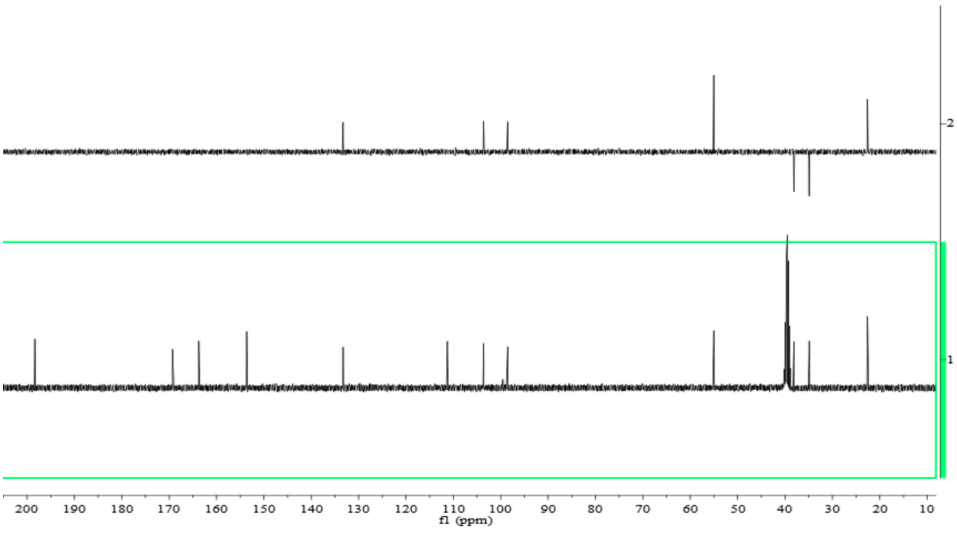

Figure S6. DEPT-135 spectrum of PA in DMSO-d_6 (150 MHz).

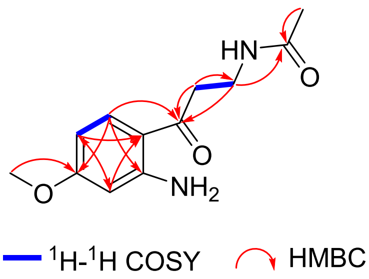

Figure S7. Key ^{1}H-^{1}H COSY and HMBC correlations of PA.

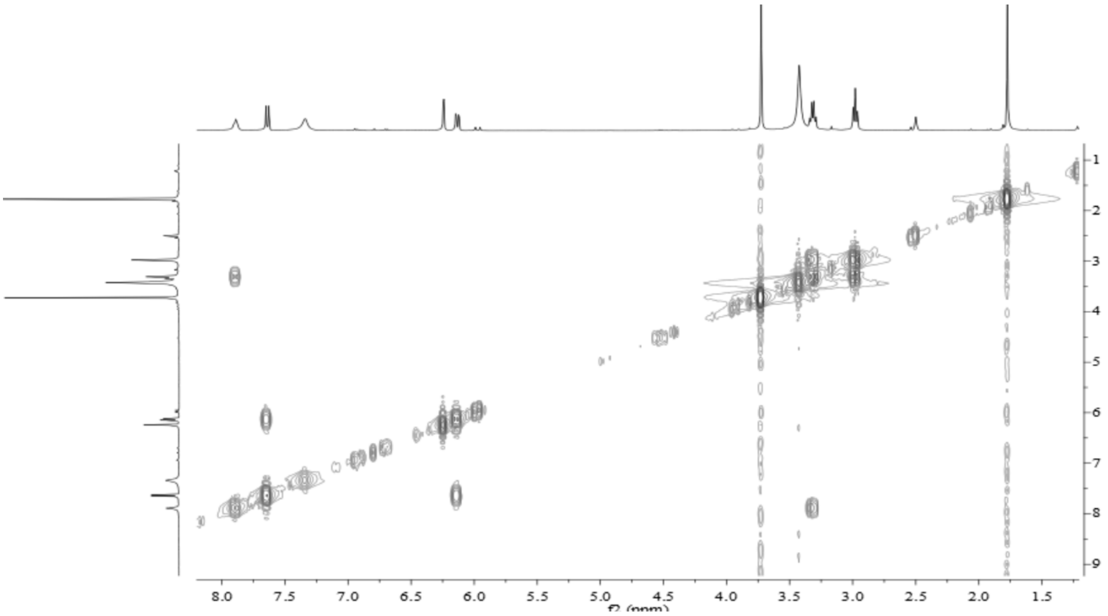

Figure S8. ^{1}H-^{1}H COSY spectrum of PA in DMSO-d_6.

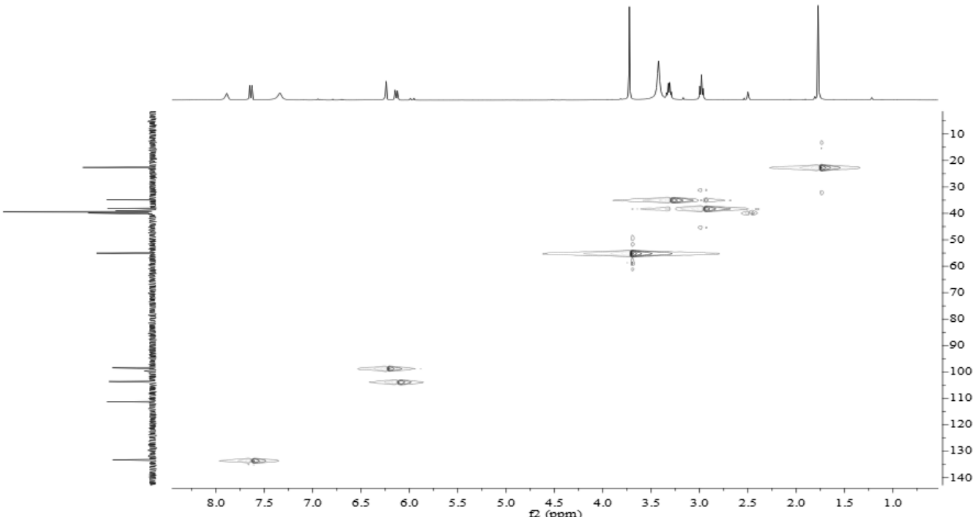

Figure S9. HSQC spectrum of PA in DMSO-d_6.

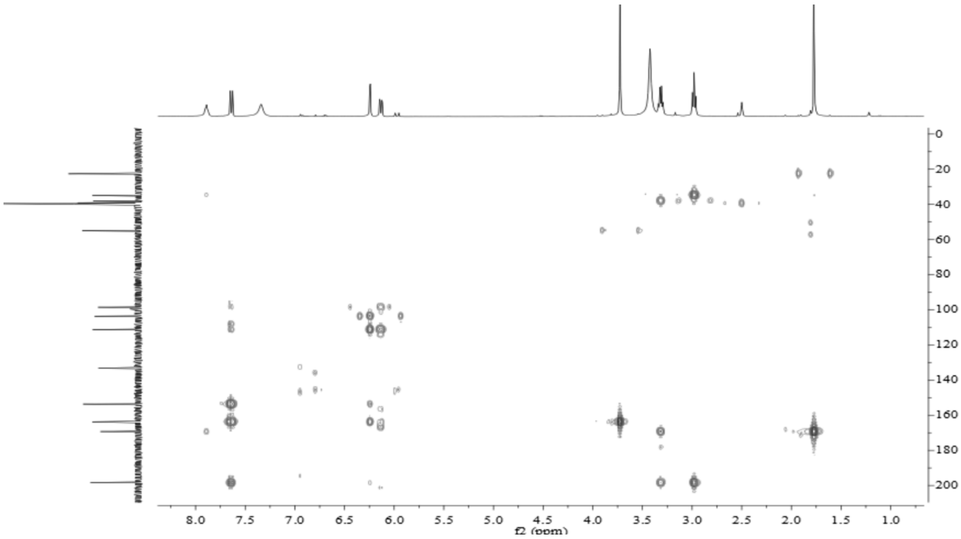

Figure S10. HMBC spectrum of PA in DMSO-d_6.

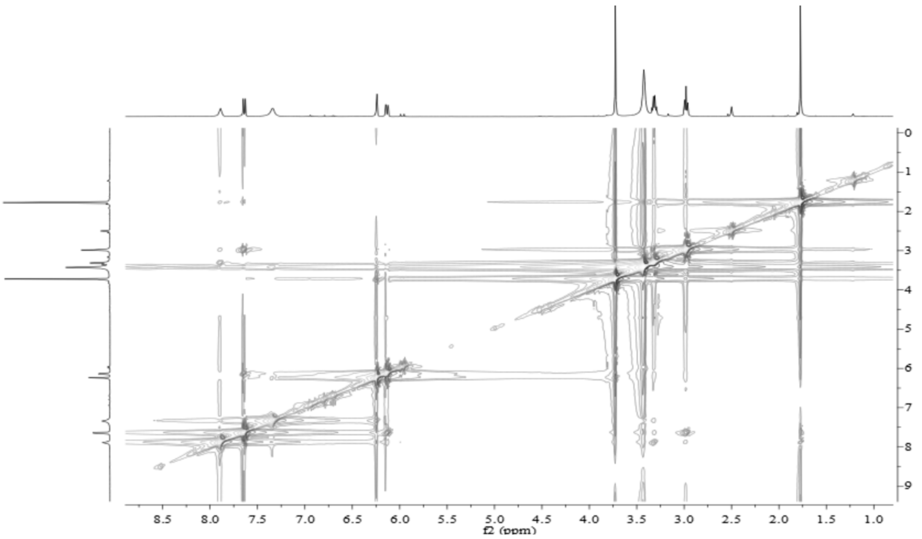

Figure S11. NOESY spectrum of PA in DMSO-d_6.

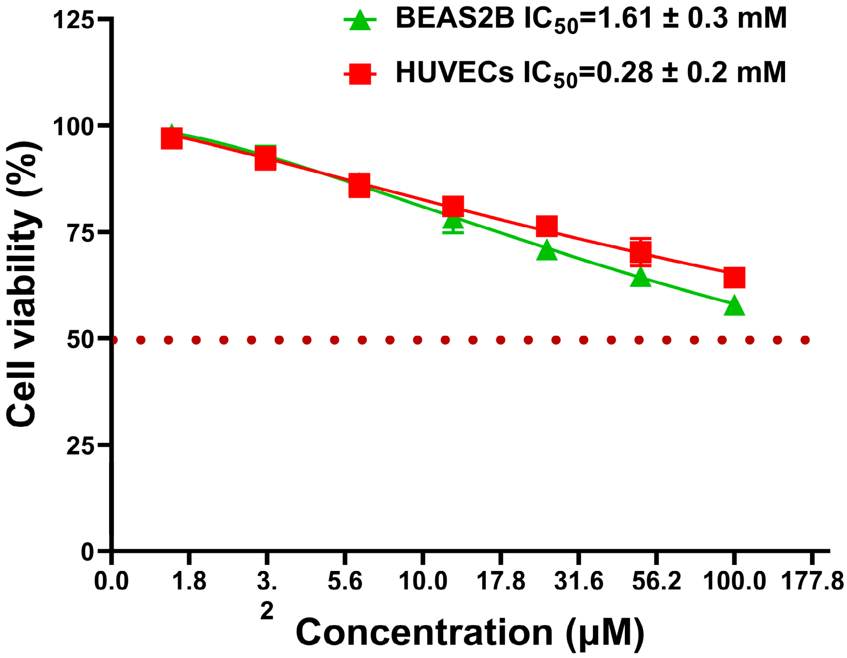

Figure S12. The toxicity of PA in BEAS-2B and HUVECs.
